# Bladder cancer course, four genetic high-risk variants, and histopathological findings

**DOI:** 10.17179/excli2023-5862

**Published:** 2023-08-18

**Authors:** Thura Kadhum, Silvia Selinski, Meinolf Blaszkewicz, Jörg Reinders, Emanuel Roth, Frank Volkert, Daniel Ovsiannikov, Oliver Moormann, Holger Gerullis, Dimitri Barski, Thomas Otto, Svetlana Höhne, Jan G. Hengstler, Klaus Golka

**Affiliations:** 1 Leibniz Research Centre for Working Environment and Human Factors at TU Dortmund (IfADo), Dortmund, Germany; 2 Specialist Clinic for Psychosomatic Rehabilitation, Mittelrhein-Klinik, Boppard - Bad Salzig, Germany; 3 Department of Urology, Evangelic Hospital, Paul-Gerhardt Foundation, Lutherstadt Wittenberg, Germany; 4 St.-Josefs-Hospital Dortmund-Hoerde, Dortmund, Germany; 5 Rheinland Klinikum Lukaskrankenhaus Neuss, Neuss, Germany

**Keywords:** muscle invasive bladder cancer (MIBC), non-muscle invasive bladder cancer (NMIBC), PSCA gene rs2294008 and rs2978974, FGFR3-TACC3 gene region rs798766, CBX6-APOBEC3A gene region rs1014971

## Abstract

Urinary bladder cancer, a smoking and occupation related disease, was subject of several genome-wide association studies (GWAS). However, studies on the course of the disease based on GWAS findings differentiating between muscle invasive bladder cancer (MIBC) and non-muscle invasive bladder cancer (NMIBC) are rare. Thus we investigated 4 single nucleotide polymorphisms (SNPs) detected in GWAS, related to the genes coding for TACC3 (transforming, acidic coiled-coil containing protein 3), for FGFR3 (fibroblast growth factor receptor 3), for PSCA (prostate stem cell antigen) and the genes coding for CBX6 (chromobox homolog 6) and APOBEC3A (apolipoprotein B mRNA editing enzyme, catalytic polypeptide-like 3A). This study is based on 712 bladder cancer patients and 875 controls from 3 different case control studies in Germany. The 4 SNPs of interest (PSCA rs2294008 and rs2978974, FGFR3-TACC3 rs798766, and CBX6-APOBEC3A rs1014971) were determined by real-time polymerase chain reaction. The distribution of the 4 SNPs does not vary significantly between cases and controls in the entire study group and in the 3 local subgroups, including two former highly industrialized areas and a region without such history. Also, no significant differences in the bladder cancer subgroups of MIBC and NMIBC were observed. The 4 investigated SNPs do not noticeably contribute differently to the bladder cancer risk for the bladder cancer subgroups of MIBC and NMIBC.

## Introduction

Bladder cancer (BC) is the 4^th^ common cancer in males and the 14^th^ common cancer in females in Germany with an estimated 22.430 men and 7.100 women newly diagnosed in 2014 (Robert Koch Institute, 2018[38]). World-wide, it is the 6^th^ common cancer in males and the 18^th^ common cancer in females with an estimated 440.864 men and 132.414 women newly diagnosed in 2020 (Sung et al., 2021[45]). This smoking and occupation related disease was a subject of several genome-wide association studies (GWAS; overview in Selinski, 2017[42]; de Maturana et al., 2018[5]). Furthermore, a considerable number of studies investigating the impact of genetically based risk factors on bladder cancer was performed (overview: de Maturana et al., 2018[5]; Golka et al., 2011[11]). Regarding the impact on the course of the disease, multiple loci have been identified, aiming at different endpoints, e.g., T category, grade, recurrence, and mortality yet demonstrating little consensus (Lipunova et al., 2019[28]).

Thus, we investigated 4 single nucleotide polymorphisms (SNPs) detected in GWAS (Kiemeney et al., 2010[18]; Rothman et al., 2010[40]; Wu et al., 2009[52]; Fu et al., 2012[7]) related to the region of the genes coding for TACC3 and for FGFR3 (transforming, acidic coiled-coil containing protein - fibroblast growth factor receptor 3) located on 4p16.3, the gene coding for PSCA (prostate stem cell antigen) located on 8q24.3, and the region of the genes coding for CBX6 (chromobox homolog 6) and for APOBEC3A (apolipoprotein B mRNA editing enzyme, catalytic polypeptide-like 3A) located on 22q13.1. Furthermore, T category and grading in muscle invasive bladder cancer (MIBC) and non-muscle invasive bladder cancer (NMIBC) and the course of the disease, i.e., the number of the relapses and their histopathological findings, were observed.

The region of the genes coding for TACC3 (transforming, acidic coiled-coil containing protein 3) and for FGFR3 (fibroblast growth factor receptor 3) is located on 4p16.3, a known Huntington disease region (Lee et al., 2012[23]). The reference SNP rs798766(T) is associated with bladder cancer risk (Kiemeney et al., 2010[18]; Meng et al., 2017[32]). rs798766 is located in an intron of TACC3, 70 kb from FGFR3 (Kiemeney et al., 2010[18]). FGFR3 belongs to the family of tyrosine kinases. FGFR3 signaling is involved in development, differentiation, cell survival, migration, angiogenesis, and carcinogenesis (Turner and Grose, 2010[46]). FGFR3 mutations in bladder cancer are mostly activating, followed by gene rearrangements and amplification (Cancer Genome Atlas Research Network, 2014[1]; Helsten et al., 2016[13]). They are mainly detected in genetically stable bladder cancer (van Rhijn et al., 2003[47]) and have been associated with progression in bladder cancer (Parker et al., 2014[36]). FGFR3 gene rearrangements generate constitutively activated and oncogenic FGFR3 kinase protein products, and cellular dependence on these drivers confers sensitivity to selective FGFR inhibition (Williams et al., 2013[51]; Wu et al., 2013[53]; Kim et al., 2018[20]). FGFR alterations are targeted in clinical routine already by the kinase inhibitor erdafitinib, which was approved by FDA in April 2019 (Loriot et al., 2019[30]). Most recently, it was shown in a phase 3 study that erdafitinib is superior to chemotherapy in the therapy of advanced or metastatic bladder cancer in patients with unresectable, advanced or metastatic urothelial cancer and select FGFR3/2 receptor alterations (mutations/fusions) (Loriot et al., 2023[29]) According to the TCGA data base, 15.43 % (29 out of 188) bladder cancer cases provided somatic mutations in their tumors (NIH, 2023[34]).

PSCA (prostate stem cell antigen) was initially identified as a prostate-specific cell-surface antigen (Reiter et al., 1998[37]), but later found to be expressed in many human tissues (Fu et al., 2012[7]). The gene coding for PSCA is located on 8q24.3. This gene encodes a glycosylphosphatidylinositol-anchored cell membrane glycoprotein. It is highly expressed in the prostate and, to lesser extent, in the bladder, placenta, colon, kidney, and stomach. It is up-regulated in many prostate cancers and expressed in cancers of the bladder and pancreas. Its polymorphisms result in an upstream start codon in some individuals (Weizmann Institute of Science, 2022[50]). rs2978974 is located 10 kb upstream of rs2294008, within an alternative untranslated first exon of PSCA (Fu et al., 2012[7]). The variant rs2294008(T) (Wu et al., 2009[52]; Fu et al., 2012[7]; Deng et al., 2019[6]; Cui et al., 2019[4]) is associated with bladder cancer risk. In a recent meta-analysis, rs2294008 was associated with bladder cancer (OR = 1.15, 95 % CI 1.11-1.18) (Cui et al., 2019[4]). Furthermore, this SNP was strongly associated with gastric cancer (OR = 1.32, 95 % CI 1.27-1.39). rs2978974 was significant, but to a clearly lower extent, associated with bladder cancer (OR 1.09; 95 % CI 1.03-1.15) (Cui et al., 2019[4]). It is noteworthy, that Fu et al. (2012[7]) reported a significant multiplicative interaction between these two SNPs (P = 0.035). The non-risk allele G of rs2978974 showed strong interaction with nuclear proteins from five cell lines tested, implying a regulatory function. Thus, Fu et al. (2012[7]) concluded that a joint effect of the two PSCA SNPs may be important for bladder cancer susceptibility. According to the TCGA data base, 0.46 % (1 out of 216) bladder cancer cases provided somatic mutations in their tumors (NIH, 2023[34]).

The gene region coding for CBX6 (chromobox homolog 6) and for APOBEC3A (apolipoprotein B mRNA editing enzyme, catalytic polypeptide-like 3A) is located on 22q13.1. This locus is located in a non-genic region approximately 25 kb centromeric of APOBEC3A and 64 kb telomeric of CBX6 (Rothman et al., 2010[40]). APOBEC is a protein family encoded by eleven genes. Subtypes of APOBEC3 can cause specific mutations in RNA and DNA at distinct preferred nucleotide contexts in human cancer (Cao and Wu, 2018[2]). APOBEC enzymes are a major source of mutation in bladder cancer. Tumors enriched for APOBEC mutagenesis have better survival (Glaser et al., 2017[8]). CBX6 is a protein coding gene. It is a component of a Polycomb Group (PcG) multiprotein PRC1 (polycomb repressive complex 1)-like complex, a complex class required to maintain the transcriptionally repressive state of many genes, including Hox genes, throughout development (Vandamme et al., 2011[48]). PcG PRC1 complex acts via chromatin remodeling and modification of histones; it mediates monoubiquitination of histone H2A 'Lys-119', rendering chromatin heritably changed in its expressibility (Weizmann Institute of Science, 2022[49]). The variant rs1014971(T) is associated with bladder cancer risk (de Maturana et al., 2018[5]; Rothman et al., 2010[40]). CBX gene variants are also associated with risk of other cancers (Li et al., 2020[25]; Lin et al., 2020[26]; Xie et al., 2020[54]). According to the TCGA data base, 0.74 % (3 out of 408) bladder cancer cases provided somatic mutations in APOBEC3A. Likewise, 0.74 % (3 out of 408) bladder cancer cases provided somatic mutations in CBX6 (NIH, 2023[34]).

## Materials and Methods

This study is based on 712 bladder cancer patients and 875 controls from 3 different case control studies: Evangelic Hospital, Paul-Gerhardt Foundation Lutherstadt Wittenberg, Lutherstadt Wittenberg, Germany (203 cases and 210 controls (Roth et al., 2012[39])), St.-Josefs-Hospital Dortmund-Hoerde, Dortmund, Germany (156 cases and 237 controls (Ovsiannikov et al., 2012[35])), and Rheinland Klinikum Lukaskrankenhaus Neuss, Neuss, Germany (353 cases and 428 controls (Höhne et al., 2017[14])). Controls were from the same respective hospitals without a known history of cancer. Enrolment of patients was as follows: Lutherstadt Wittenberg from December 1995 to January 1999, Dortmund from July 2009 to December 2010, and Neuss from June 2009 to November 2011. Follow-up derived from medical records was as follows: Lutherstadt Wittenberg from September 2008 to June 2009, Dortmund from May 2012 to August 2012, Neuss from July 2013 to February 2014 (Selinski et al. 2016[44]; Roth et al., 2012[39]).

### Analytical methods

DNA of cases and controls was isolated from venous blood samples according to standard methods in the Dortmund Institute (Selinski et al., 2017[43]). The 4 SNPs of interest (PSCA rs2294008 and rs2978974, gene region FGFR3-TACC3 rs798766, and gene region CBX6-APOBEC3A rs1014971) were detected by real-time polymerase chain reaction (rt-PCR) using a TaqMan^®^ assay (Saravana Devi et al., 2008[41]; Selinski et al., 2017[43]). The missings for the detected SNPs for cases and controls respectively were as follows: 0 and 0 for rs2294008, 2 and 1 for rs2978974, 0 and 0 for rs798766, and 0 and 4 for rs1014971. 

### Statistical methods

Hardy-Weinberg Equilibrium was checked using a chi-squared test with one degree of freedom. Chi-squared tests were used testing differences between categorial variables, logistic regression was used to compute Likelihood Ratio and Wald tests as well as odds ratios (OR) and 95 % confidence intervals (95 % CI). The analysis was conducted searching for differences between cases vs controls, recurrent vs non-recurrent and MIBC vs NMIBC and stratified for gender, invasiveness, and smoking habits. False discovery rate (FDR) was used for multiple testing correction.

The studies in the 3 departments of urology were approved by the ethics committee of the Dortmund Institute (24/2009, 31/2009, 50/2011, 74/2014) and by the Institutional Review Board.

## Results

The total study group contains 3 subgroups from different regions in Germany with comparable characteristics regarding gender and age among cases as well as among controls. In bladder cancer patients, the portions of the described characteristics were as follows: Total study group: 79 % male, median age 70.5 years. Wittenberg: 86 % male, median age 65.4 years. Dortmund: 75 % male, median age 71.0 years. Neuss: 77 % male, median age 73.2 years. Detailed information, including that of the controls is presented in Table 1(Tab. 1) and 2(Tab. 2).

A description of the clinical parameters of the study group is presented in Table 3a, b(Tab. 3). As expected, in the combined group of muscle invasive bladder cancer (MIBC) and non-muscle invasive bladder cancer (NMIBC), significant differences were observed regarding the smoking habits. A total of 16 % of the male patients, in contrast to 49 % of the female patients were non-smokers (p <0.0001). The portions of the different T categories did not differ significantly between male and female patients.

Male patients showed a higher percentage of NMIBC (82 % vs 79 %), whereas female patients showed a higher percentage of MIBC (21 % vs 18 %). Grading differed significantly between the genders in 2 subgroups (Table 3b(Tab. 3)). There was no significant gender-related difference in the distribution of NMIBC and MIBC (Table 3a(Tab. 3)). A stratification of the clinical parameters for NMIBC and MIBC is presented in Table 3b(Tab. 3). 

The frequency of the 4 investigated SNPs in the combined group of NMIBC and MIBC cases and in controls, additionally stratified for the 3 local subgroups, is presented in Table 4(Tab. 4). 

The distribution of the 4 investigated SNPs, stratified for NMIBC and MIBC cases does not vary significantly between the 3 local subgroups, including two formerly highly industrialized areas with industries associated with elevated bladder cancer risk (Dortmund (Ovsiannikov et al., 2012[35]) and Lutherstadt Wittenberg (Roth et al., 2012[39])) and a region with no industries with known bladder cancer risk (Neuss (Höhne et al., 2017[14])). No significant differences between cases and controls and between the subgroups of NMIBC and MIBC bladder cancer were observed (Table 5(Tab. 5)). 

The subgroup of cases with progression stratified for the histopathological findings for T category and grading are presented in Table 6(Tab. 6). Due to the small number of cases with progression, a statistical evaluation of the impact of the 4 investigated SNPs was not performed.

## Discussion

Since the first papers on the impact of polymorphism on the metabolism of the antitubercular agent isoniazid (INH) in the 1950s (Hughes et al., 1954[15]; Mitchell et al., 1957[33]), a considerable number of studies have been published on the impact of genetic polymorphisms on bladder cancer risk (overview: Golka et al., 2011[11]). However, with the first paper on genome-wide association studies on bladder cancer risk (Kiemeney et al., 2008[19]), a new era started, showing the impact of genetics on bladder cancer of loci, mostly not associated with bladder cancer before (Golka et al., 2011[11]).

To date, about 40 single nucleotide polymorphisms are shown to be associated with bladder cancer risk, mostly within the range between 1.1 and 1.5, mostly identified and/or confirmed in GWAS (Selinski, 2017[43]; de Maturana et al., 2018[5]; Koutros et al., 2023[22]). This is much lower than the risk conferred by the portion of N-acetyltransferase 2 (NAT2) slow acetylators reported in the first studies on bladder cancer risk due to high exposure to highly carcinogenic aromatic amines in the chemical industry at that time (Lewalter and Miksche, 1992[24]; Cartwright et al., 1982[3]; Golka et al., 2002[10]) or conferred by the portion of GSTM1 negatives reported formerly in highly industrialized areas (Golka et al., 1998[9]; Hung et al., 2004[16]). 

The first study on variants detected in GWAS on bladder cancer course was published by Roth et al. (2012[39]), showing an association of longer relapse-free survival in GSTT1 positives (HR = 0.60, 95 % CI = 0.42-0.87) in the formerly highly industrialized area of Lutherstadt Wittenberg. GSTM1 negatives tended to be associated with a better prognosis, whereas the NAT2 risk alleles rs710521(A) and rs9642880(T), detected in the first GWAS on bladder cancer (Kiemeney et al., 2008[19]) tended to be associated with a poor prognosis. Since then, only few studies on the impact of genetic polymorphisms detected in GWAS on the course of the bladder cancer disease have been conducted (Grotenhuis et al., 2014[12]; Lipunova et al., 2019[27]), most possibly due to the enormous logistic effort. Suitable candidates for an impact on the course of the disease are those genetic polymorphisms and/or loci, which have a proven influence on bladder cancer risk (Roth et al., 2012[39]). Another promising approach is the investigation of the effect of SNP combinations or, even better, a large panel of SNPs as recently applied for the determination of the risk to contract bladder cancer (Koutros et al., 2023[22]). Although our group has experience with the impact of SNP combinations on bladder cancer risk regarding smoking habits (Selinski et al., 2017[43]), we decided not to apply this approach for this study because the available case numbers are by far too small when two or more polymorphisms are combined.

It is now established for a long time that bladder cancer is no more one tumor entity, but two: Non-muscle invasive bladder cancer (NMIBC) and muscle-invasive bladder cancer (MIBC) (Knowles and Hurst, 2015[21]). It may be noted, that genetic analysis has resulted in a new biological classification for MIBC discriminating 6 different subtypes (Kamoun et al., 2020[17]). Similar activities for NMIBC are undergoing (Marzouka et al., 2022[31]). Due to the very different course of these two tumor entities, we decided to investigate the impact on the subgroups of NMIBC and the clinically much more important MIBC separately in larger study group now including in addition to Roth et al. (2012[39]), patients from two other studies (Ovsiannikov et al., 2012[35]; Höhne et al., 2017[14]). The 4 investigated SNPs showed no noticeable differences in the frequency between the NMIBC and MIBC subgroups. This is in line with the findings of Grotenhuis et al. (2014[12]) for PSCA gene related rs2294008, TACC3 - FGFR gene region related rs798766, and CBX6 - APOBEC3A gene region related rs1014971. This may be due to the minimal impact of a single SNP. Thus, in future studies an impact of SNP combinations should be investigated on large study groups, as this procedure had successfully shown that a 4 SNP combination confers increased bladder cancer risk particularly in never smokers (Selinski et al., 2017[43]). Recently, this approach was applied by a GWAS using a 24 SNP marker panel (Koutros et al., 2023[22]). Another promising approach would be to investigate the impact of selected SNPs and/or their combinations in the clinically relevant group of symptomatic patients showing micro and/or gross hematuria.

## Declaration

### Author contributions

Conceptualization T.K., S.S., T.O., J.G.H., and K.G., methodology S.S., M.B., and J.R., software S.S., laboratory analysis M.B., J.R., data analysis T.K., S.S., investigation T.K., S.S., T.O., and K.G., resources F.V., O.M., T.O., and J.G.H., data curation E.R., D.O., H.G., D.B., S.H., and S.S., writing-original draft preparation T.K., S.S., M.B., and K.G., writing-review and editing T.K., T.O., and K.G,. visualization T.K., K.G., supervision F.V., O.M., T.O., and J.G.H, project administration J.G.H., K.G. All authors have read and agreed to the published version of the manuscript. 

### Funding

This research received no external funding. 

### Institutional review board statement

The study was conducted in accordance with the Declaration of Helsinki and approved by the Institutional Review Board and by the Ethics Committee of Leibniz Research Centre for Working Environment and Human Factors at TU Dortmund (IfADo).

### Informed consent statement

Informed consent was obtained from all subjects involved in the study.

### Data availability statement

The data are available on reasonable request from the corresponding author.

### Conflicts of interest

The authors declare no conflict of interest.

## Figures and Tables

**Table 1:**
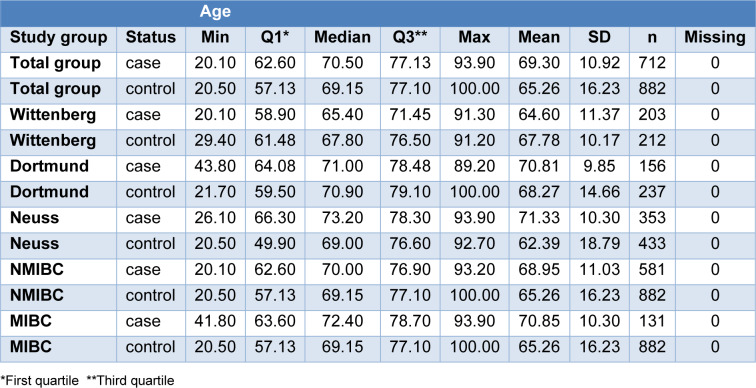
Age distribution in the total study group of bladder cancer cases and the subgroups of non-muscle invasive bladder cancer (NMIBC) and muscle-invasive bladder cancer (MIBC), and controls

**Table 2:**
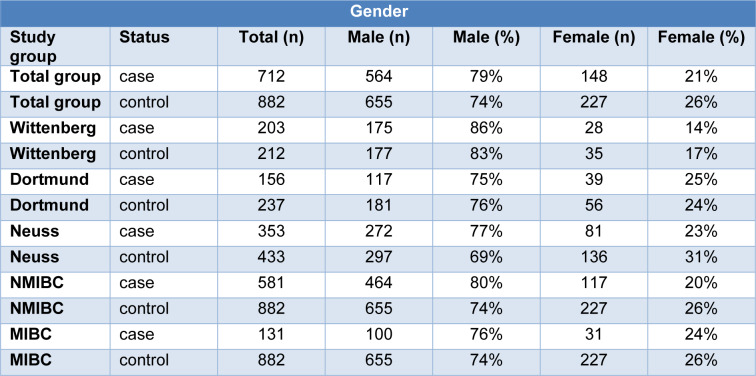
Gender distribution in the total study group of bladder cancer cases and the subgroups of non-muscle invasive bladder cancer (NMIBC) and muscle-invasive bladder cancer (MIBC), and controls

**Table 3:**
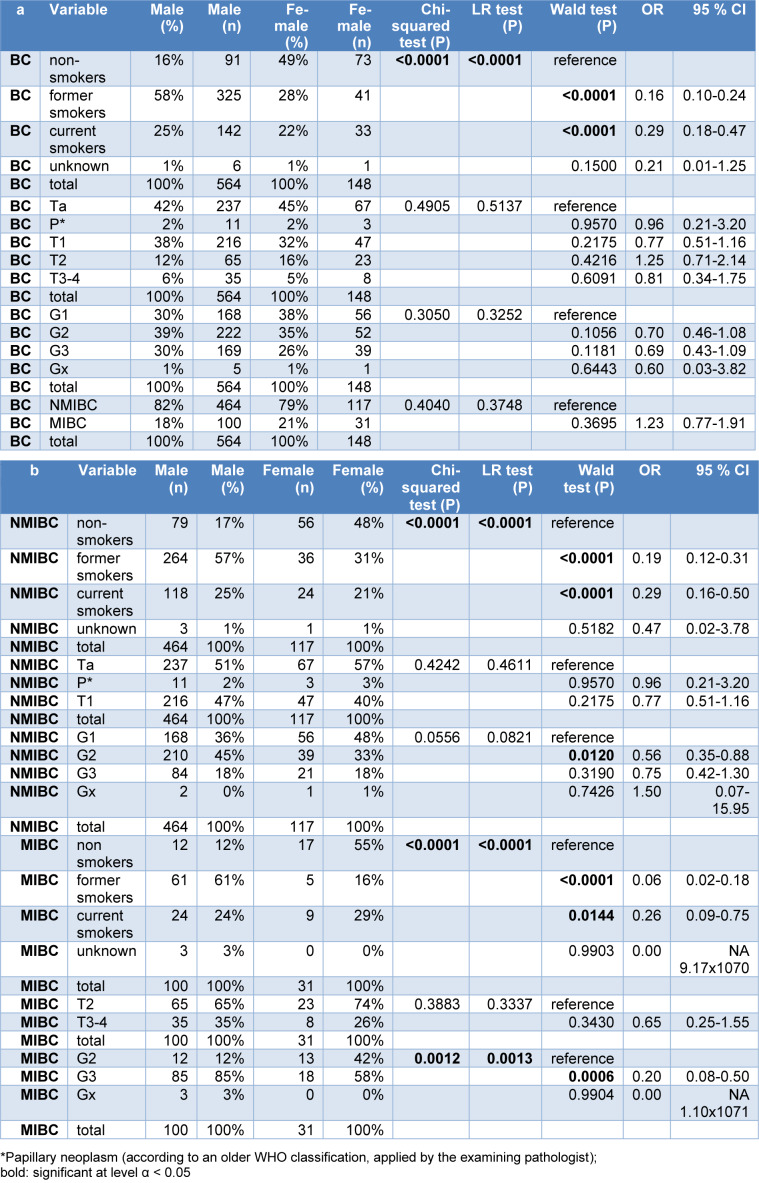
Description of the total study group of bladder cancer (BC) cases (a) and the subgroups of non-muscle invasive bladder cancer (NMIBC) and muscle-invasive bladder cancer (MIBC), stratified for gender, smoking habits, T category, and grading (b).

**Table 4:**
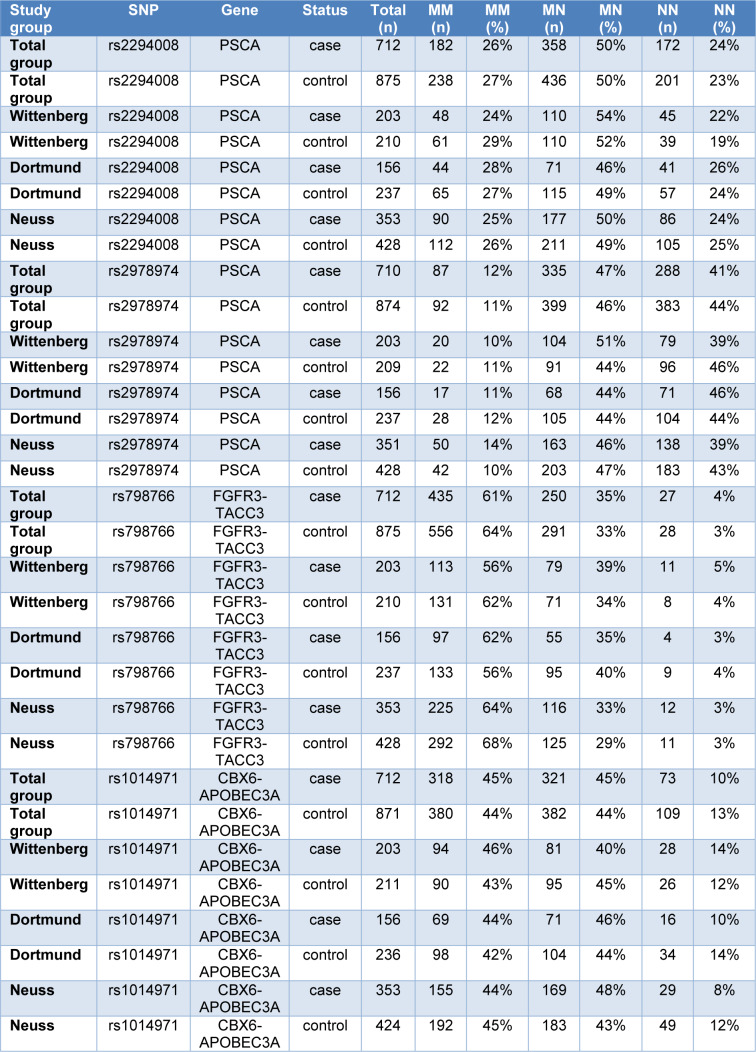
Frequency of the double mutated (MM), heterozygous (MN) and double native (NN) status of the 4 investigated single nucleotide polymorphisms (SNPs) in the total group of bladder cancer cases and in controls stratified for the 3 local subgroups

**Table 5:**
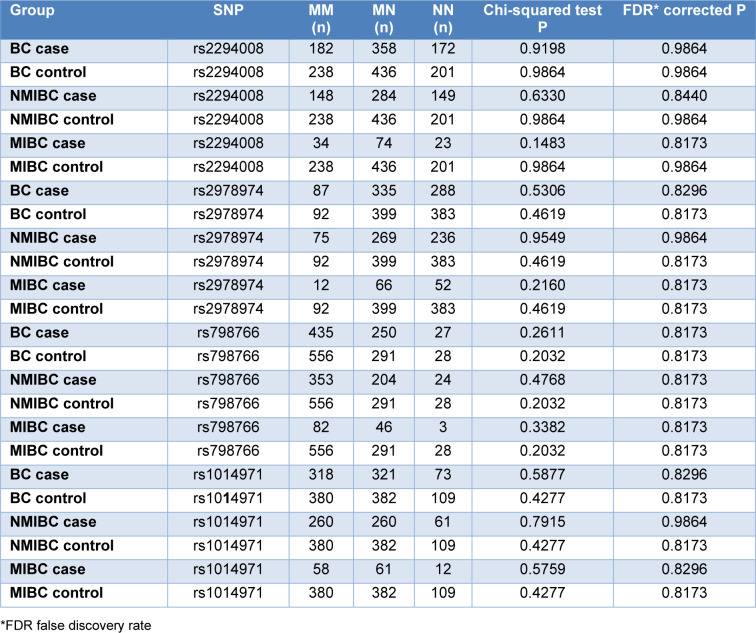
Frequency of double mutated (MM), heterozygous (MN) and double native (NN) status of the 4 investigated single nucleotide polymorphisms (SNPs) in bladder cancer (BC) cases, controls and in the BC cases stratified for non-muscle invasive (NMIBC) and muscle invasive (MIBC) bladder cancer

**Table 6:**
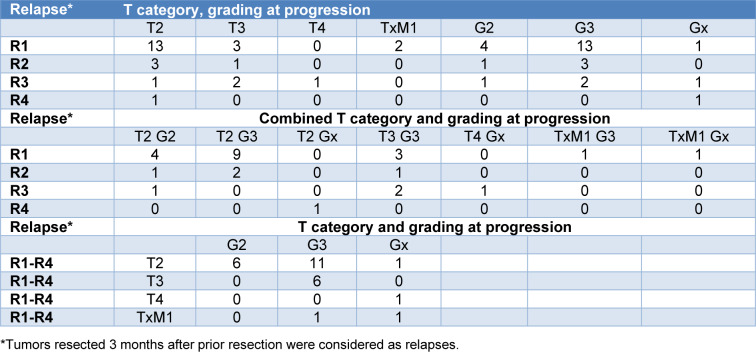
T category, staging and grading at progression in the investigated bladder cancer cases
